# Influence of circumferential ankle pressure of shoe collar on the kinematics, dynamic stability, electromyography, and plantar pressure during normal walking

**DOI:** 10.1371/journal.pone.0281684

**Published:** 2023-02-10

**Authors:** Alireza Nasirzadeh, Seung-Tae Yang, Juseok Yun, Jaeha Yang, Young Yoon Bae, Juyeon Park, Jooeun Ahn, Giuk Lee

**Affiliations:** 1 Department of Mechanical Engineering, Chung-Ang University, Seoul, Republic of Korea; 2 Department of Textiles, Merchandising and Fashion Design, Seoul National University, Seoul, Republic of Korea; 3 Department of Physical Education, Seoul National University, Seoul, Republic of Korea; Ningbo University, CHINA

## Abstract

**Background:**

The shoe’s collar plays a significant role in supporting the ankle during walking. Since the protective effect of the collar requires the circular embracing of the ankle and shank, a stiffer collar might be involved with increased circumferential ankle pressure (CAP). It is not clear how collar CAP affects walking performance. Therefore, this study was aimed at examining the influence of the collar CAP on the kinematics, dynamic stability, electromyography (EMG), and plantar pressure during normal walking.

**Method:**

Sixteen healthy male participants walked on a treadmill while wearing a custom-designed high-collar shoe with 10 (low), 30 (medium), and 60 mmHg (high) CAP conditions, and the joint angles, dynamic stability index, EMG, and plantar pressure were measured.

**Result:**

While the low CAP condition did not affect the ankle range of motion (ROM), The high CAP condition restricted both the ankle sagittal and frontal ROM, whereas the medium CAP condition limited only the ankle frontal ROM. The knee and hip ROM did not differ between conditions. The dynamic stability for the high and medium CAP cases was comparable but significantly higher than that of the low CAP condition. The ankle muscle activity and corresponding co-contraction increased with increasing CAP for gastrocnemius medialis (GM), soleus (SOL), peroneus longus (PL), tibialis anterior (TA) muscles in the weight acceptance and push-off phases but not in the single limb support. Knee muscle activity, including vastus lateralis (VL) and semitendinosus (SEMI) was similar between all conditions. A higher relative pressure was observed under the lateral aspect of the heel when walking in the high CAP condition.

**Conclusion:**

The results suggest that a high-collar shoe with a high CAP may not be an appropriate choice for walking owing to the injury risk factors and limited walking efficiency. A medium CAP is associated with certain advantages and, thus, a superior choice for high-collar shoe design.

## Introduction

During walking, footwear serves as the interface between the feet and the walking surface and transfers the power produced by the musculoskeletal system to the supporting surface. Consequently, the footwear characteristics influence the somatosensory feedbacks and dynamic postural stability, which affect the risk of falling [1]. An appropriate shoe enhances postural stability, decreases sway, and prevents falls by improving the base of support (BOS) and somatosensation [2]. In other words, the shoe design must be optimized to enhance walking performance.

The collar design is a critical shoe feature that has been the subject of several biomechanical walking investigations. A high-collar shoe provides external support and stability to the ankle joint and can potentially protect it from injuries [3]. In general, lateral ankle sprains commonly occur while walking and hiking [4] or engaging in sports activities [4]. A high-collar shoe can restrict excessive ankle inversion, which is the most prevalent mechanism for lateral ankle sprains [4]. According to gait analysis studies, wearing high-collar shoes during walking may influence the lower limb joints’ range of motion (ROM) [5–7], shock absorption and power generation [5, 6], and muscular activity [5, 8, 9]. It seems that a stiffer collar restricts ankle joint ROM, and muscle activity necessary to stabilize the ankle joint is likely to decrease when the demand placed on the lower limb is reduced, perhaps due to the superior support provided by a stiffer high-collar [9]. The amount of support a shoe collar can provide to the ankle can be manipulated by tuning the collar stiffness. The shoe collar stiffness can significantly influence the capacity of the ankle joint to adapt the foot to the supporting ground via the subtalar joint, which may impair gait stability [10]. A shoe with a softer collar enables a wider ROM in the ankle joint, resulting in more power generation in the ankle joint during the push-off, as well as increased step length and gait velocity [7]. In contrast, wearing a shoe with a rigid collar may lead to reduced power generation at the ankle joint [11], decreased gait efficiency, and promotion of knee joint loading, which can cause early fatigue of the knee muscles during walking [5]. Considering these aspects, a shoe with a softer collar may be considered a superior choice for walking. However, a shoe with considerable collar stiffness might be required to protect the ankle joint from injuries, mainly caused by excessive inversion [12]. Thus, the appropriate collar stiffness must be identified.

Many researchers investigated the protective effect of high-collar shoes during walking and other activities by considering the collar height [13–15] and stiffness [3, 5–7, 13]. Circumferential ankle pressure (CAP) is another notable collar property that directly influences the protective effect and comfort level. Because the shoe collar’s protective action requires a circular embracing of the ankle and shank, there is a direct relationship between collar-provided support and CAP. In general, deficits in ankle proprioception are predictive of ankle injuries [16]. Increasing the CAP can enhance proprioceptive acuity and active stiffness in the ankle, therefore increasing postural stability [17]. However, a high CAP may lead to ankle restriction, which can decrease the functional ROM and power generation of the ankle joint [5]. A higher ankle restriction may also deteriorate walking performance and introduce an undesired compensatory mechanism at the knee and hip joint level [5, 6], potentially leading to other complications. Therefore, the amount of CAP that the shoe collar apples to the ankle influence the walking biomechanics, and the shoe collar CAP must be set to ensure a balance between protection and performance. To the best of our knowledge, none of the existing studies have investigated the influence of a high-collar shoe CAP on normal walking from a biomechanical perspective. Therefore, this study aimed to examine the influence of the CAP associated with a custom-made high-collar shoe on kinematics, dynamic stability, electromyography (EMG), and plantar pressure during normal walking. We hypothesize that Increasing CAP would restrict ankle joint movement, improve dynamic stability, decrease EMG, and change plantar pressure pattern.

## Materials and methods

### Participants

A power calculation was done to determine the sample size using the data from a previous studies [5, 6]. A sample size calculation indicated that for 80% power and a *P* value of 0.05, at least 10 participants were required. To mitigate the possible effect of subject dropout, a total of 16 participants were considered to be sufficient. Sixteen healthy active males (age: 24.56±2.56 y; height: 1.78±0.05 m; weight: 71.25±6.12 kg; BMI: 22.42±1.62 kg/m^2^; foot size: 27.19±2.50 cm; mean ± standard deviation) from the Chung-Ang University community participated in the study. The inclusion criteria were as follows: a US shoe size of 10 (27.0–27.5 cm), no recent musculoskeletal or neuromuscular problems or injuries, and no history of surgery influencing the walking performance. An experienced biomechanist assessed the volunteers’ foot arch (using the navicular drop test; 5 mm<normal<10 mm), static rearfoot eversion (0°<normal<4°) [18], and legs, and the volunteer was excluded if they exhibited hyperpronation, genu valgum, or genu varum.

Before participation, the participants were notified of the study’s objectives and familiarized with the experimental protocol. All participants provided written consent. The study was performed following the principles outlined in the Declaration of Helsinki and approved by the Chung-Ang University ethics committee and research review board.

### Experimental procedures

#### Experimental protocol

Walking trials were performed at 1.25 m/s (4.5 km/h) on a custom-made treadmill with no inclination. After attaching markers and sensors, the participants were requested to walk on the treadmill for 5 min at a comfortable pace for warm-up. The individuals’ experience of wearing a new shoe was expected to influence their postural and dynamic responses to the footwear [19]. Therefore, we allowed participants sufficient time to adapt to the study shoe while walking. After familiarization, all participants completed three successful walking trials while wearing the custom-made high-collar shoe with low, medium, and high CAP conditions (Section 2.2.2). Participants walked for 30 s in each trial, and the CAP condition order was randomized. The participants rested for 5 min between trials while the footwear CAP was adjusted.

#### Custom-made footwear and CAP conditions

**Fig 1:**
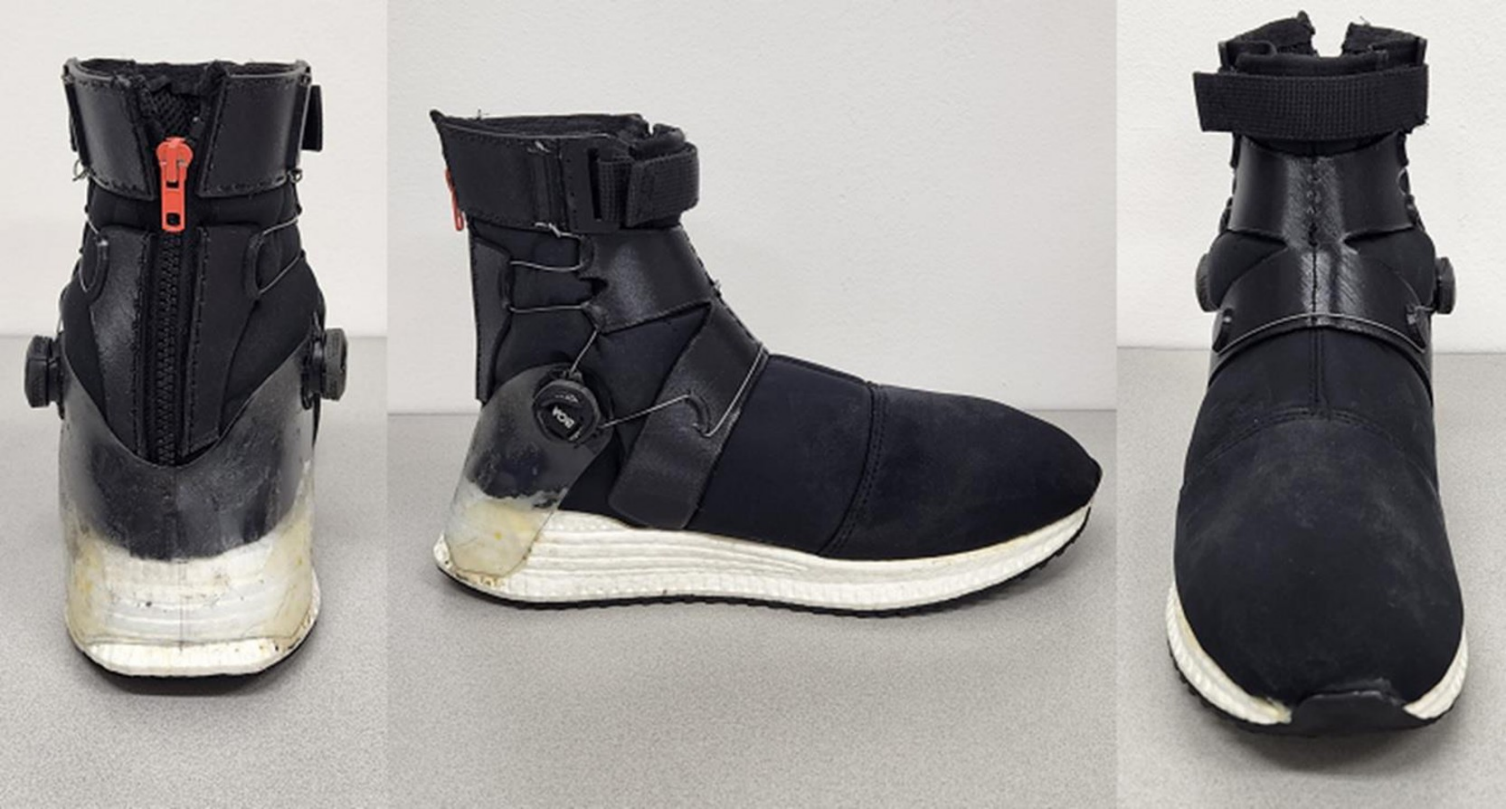
The prototype high-collar shoe was designed for this study. A wire connects two ratcheting reels to uniformly spread the pressure around the ankle. This design allows precise control over CAP by adjusting the ratcheting reels and top strap.

To adjust the CAP, we designed a high-collar shoe (mass: 405 g; size: 10 US; length: 29 cm; width: 11 cm; collar height: 17 cm; collar diameter: 10 cm; heel height: 41 mm; heel-midfoot drop: 17 mm) (Fig 1). The shoe was made of polyester mesh. This material does not get bogged down with sweat, is highly breathable, and provides high mobility. The black cover was made using Poly Lactic Acid 3D printing. The midsole and outsole were made of thermoplastic polyurethane (TPU) and rubber, respectively. The shoe had two Boa® ratcheting reels (Boa Technology Inc, USA) at the lateral sides connecting with zigzag patterned wiring passed through a top cover 3d printed from Poly Lactic Acid (PLA). This design allowed accurate and consistent adjustment of the CAP applied by the shoe collar around the ankle joint. The stiffness properties of this shoe are available elsewhere [20].

Two pressure-measurement sensors (Kikuhime, TT Medi Trade, Denmark) were used to measure and adjust the CAP. After wearing the shoe, while the participant was standing, we placed the pressure-measurement sensors directly on the medial and lateral ankle malleolus inside the shoe. Next, the shoe ratcheting reels and top strap were adjusted until both pressure sensors displayed the same values of 10, 30, and 60 mmHg, corresponding to low, medium, and high CAP conditions, respectively. These values were selected based on a pilot study in which five participants’ perceptions of the low, medium, and high CAP while walking were determined. 10-mmHg was the minimum amount that participants felt collar support, and 60-mmHg was the highest amount just before being uncomfortable. The pressure-measurement sensors were removed by unzipping the rear zipper of the shoe. Finally, to ensure the same CAP for both shoes, the perceptions of all participants were asked, and minor modifications were introduced in the case of any difference in the CAP.

### Equipment

#### Motion analysis

An eight-camera T-10 Vicon motion capture system (Oxford Metrics LTD. Oxford, UK) operating at a sampling frequency of 200 Hz was used to obtain kinematic data. We used a customized full-body Vicon Plug-in-Gait marker set (PiG-Vicon Motion Systems, Oxford, UK) with 74 light-reflective 1 cm markers and cluster markers on the femur, shank, upper arm, and forearm. During the static trial, additional calibration markers were attached to the medial aspects of the ankle, knee, elbow, and wrist joints to enhance the reliability of the joint angle data [21].

#### Muscular activity

To record the surface EMG data, we used a DELSYS Wireless EMG System (Trigno TM Wireless System, DELSYS Inc., USA) operating at a sampling frequency of 2000 Hz and synchronized with the Vicon capture system. The gastrocnemius medialis (GM), soleus (SOL), peroneus longus (PL), tibialis anterior (TA), vastus lateralis (VL), and semitendinosus (SEMI) muscles of the dominant lower limb were recorded. The dominant leg was identified as the preferred leg for kicking a ball [22], and all participants were right-footed.

Before using double-sided tape to attach the wireless EMG electrodes, the skin was carefully prepared by shaving the hair and swabbing the location with alcohol to decrease the skin impedance. The EMG electrodes were placed considering the SENIAM guideline recommendations [23]. To prevent skin movement artifacts during dynamic activity, all EMG electrodes were secured using an extra hypoallergenic tape. The same researcher placed all reflective markers and EMG electrodes. Owing to technical issues and sensor failure, the EMG data for two participants could not be used in the analysis.

Four foot switch sensors were applied to the heel and toe of each pressure insole using a fine wire and tape to identify the gait events. These sensors were connected to the four channels of the wireless EMG system. In our pilot study, we noted that these sensors did not significantly influence the plantar pressure data.

#### In-shoe pressure data

The in-shoe pressure was measured using a wireless X4 XSENSOR pressure-measurement system (XSENSOR Technology Corporation, Calgary, AB, Canada) operating at the preset sampling rate of 150 Hz. As recommended by the manufacturer, before each measurement session, a zero-pressure filter was applied while the pressure insole was placed in the shoe resting on the floor. Each wireless pressure insole was composed of 230 capacitive pressure sensors, each with a pressure range of 1–128 psi. The plantar pressure data were collected using a laptop that was not a part of the motion capture and EMG system. We used verbal commands to start and terminate data acquisition in each trial. By counting the walking cycles, we selected the exact cycles for plantar pressure analysis based on the motion capture data.

#### Data processing and analysis

Ten consecutive walking gait cycles of the dominant lower limb for each participant were selected for data processing (160 in total). These gait cycles were extracted from the middle of the walking trials to ensure that the participant’s performance was adequately adapted to the imposed condition.

The foot’s plantar surface was split into seven anatomical regions: the lateral rearfoot, medial rearfoot, midfoot, lateral forefoot, medial forefoot, hallux (big toe), and lesser toes. The peak pressure, mean pressure, and impulse (pressure–time integral) during the stance phase were calculated using the XSENSOR Foot and Gait software (XSENSOR Technology Corporation, Calgary, AB, Canada). The rearfoot mediolateral plantar pressure ratio was computed using the following formula [24]:

$$\left(\text{Medial}\;\text{rearfoot}\;\text{mean}\;\text{pressure}/\text{lateral}\;\text{rearfoot}\;\text{mean}\;\text{pressure}\right)\times 100\%$$ (1)

A smaller percentage corresponded to increasing relative lateral heel pressures, whereas a higher percentage was suggestive of increased relative pressure beneath the medial side of the heel.

A fourth-order band-pass Butterworth filter with cutoff frequencies of 50 and 450 Hz was used to filter EMG data. To reduce the noise, a fourth-order zero-phase lag Butterworth low-pass filter with a cutoff frequency of 10 Hz was used for the full-wave rectified EMG and raw kinematic data. For each subject, the EMG data for each muscle was normalized to the maximum value across all trials of the same muscle [5]. The stance phase was subdivided into three periods: weight acceptance, single limb support, and push-off. Using foot switch sensors, the timing of the three phases was identified considering the contact of the opposite limb, resulting in single and double support periods, defined as the time interval when one foot and both feet were in touch with the ground, respectively. The EMG data were integrated with respect to the phase duration to determine the level of muscle activity.

Using the normalized EMG data, the muscle co-contraction was quantified for the defined muscle pairs at the ankle and knee joints using the co-contraction Index (CI) equation introduced by Rudolph et al. [25]:

$$\text{CI}={\text{EMG}}_{\text{LA}}/\;{\text{EMG}}_{\text{HA}}\times \left({\text{EMG}}_{\text{LA}}+{\text{EMG}}_{\text{HA}}\right)$$ (2)

where EMG_LA_ and EMG_HA_ represent the EMG data of the less and more active muscles in the pairing, respectively, to avoid dividing-by-zero errors. In general, a higher CI indicates a high activation level of both muscles over a long period. In contrast, a low CI is suggestive of either low-level activation of both muscles or higher activity of one muscle with lower activation of the other paired muscle [25]. The GM, SOL, and PL muscles were paired with the TA for the ankle joint, and the VL and SEMI were considered agonist-antagonist pairs for the knee joint. These muscle pairs were selected considering their functional contributions to joint stabilization and enhancement in the bone-to-bone contact force [8]. The averaged CI over the weight acceptance, single limb support, and push-off intervals were evaluated.

**Fig 2:**
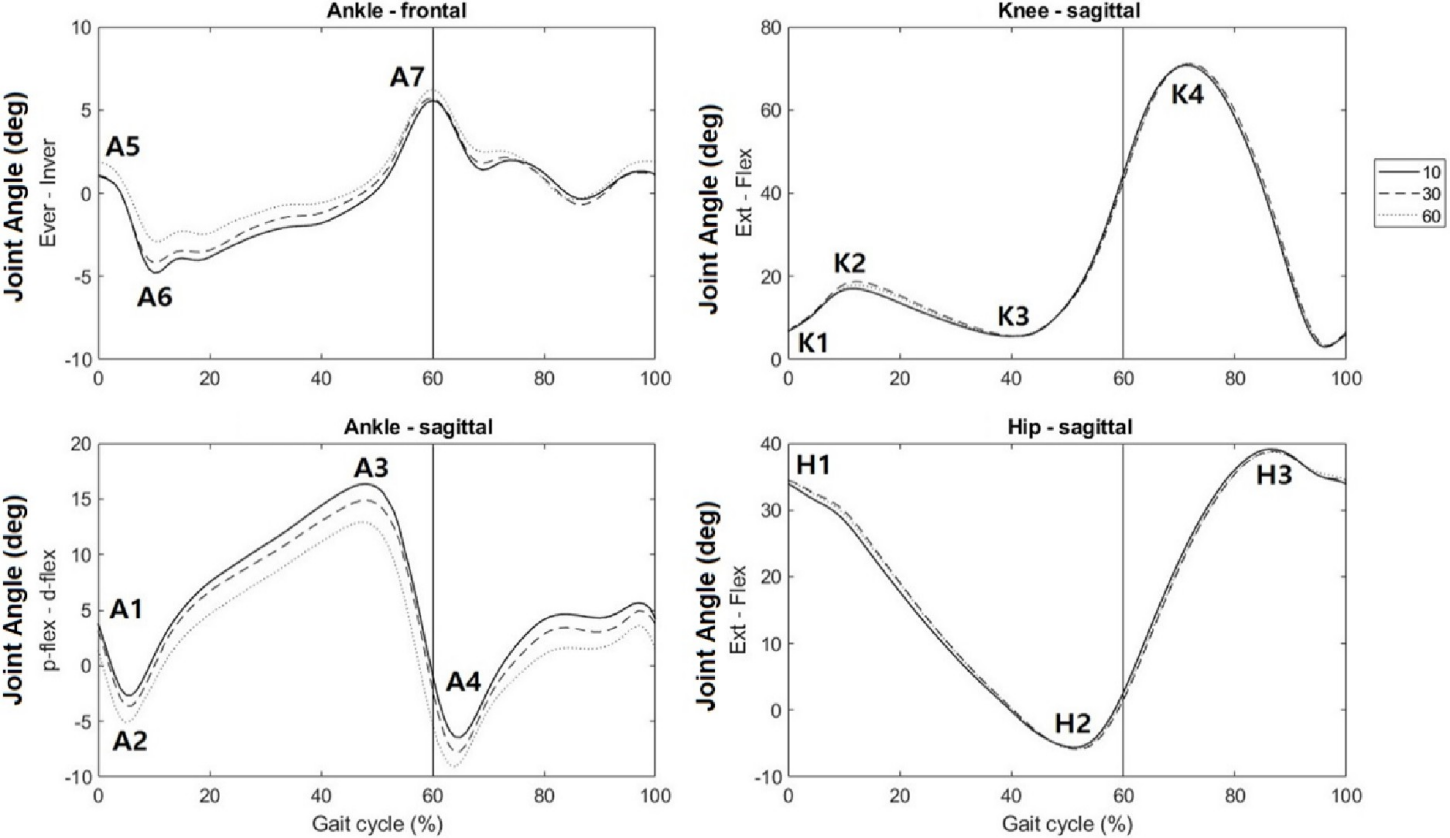
Averaged ankle, knee, and hip joints angle during walking with three shoe conditions.

The hip, knee, and ankle joint angles for the dominant lower limb during ten consecutive strides were calculated using the Visual3D software (CMotion Inc., Rockville, MD). According to the conventions of the International Society of Biomechanics, extension at the hip and knee and plantarflexion at the ankle were assigned positive directions [26]. We calculated the ROM and angular parameters for the ankle (A1: heel strike sagittal, A2: maximum plantar flexion during stance, A3: maximum dorsiflexion during stance, A4: maximum plantar flexion during swing, A5: heel strike frontal, A6: maximum eversion, A7: maximum inversion), knee (K1: heel strike, K2: maximum flexion during stance, K3: maximum extension during stance, K4: maximum flexion during the swing), and hip (H1: heel strike, H2: maximum extension, H3: maximum flexion during the swing) (Fig 2).

A 15-segment model generated in Visual3D software was used to determine the body center of mass (COM) trajectory. The lateral margin of the BOS was estimated using the fifth metatarsal marker (spatial location) and foot anthropometrics (marker position relative to borders of the foot). With reference to the work of Menant et al. [1], the mediolateral dynamic stability index during the single-limb support was calculated as the minimum distance between the mediolateral location of the COM transverse plane projection and lateral margin of the BOS, normalized to the step width. All mathematical calculations were performed using MATLAB (The MathWorks Inc., Natick, MA, USA).

### Statistical analysis

The results were expressed as the mean ± standard deviation. The Shapiro–Wilk test was performed to examine the normality of distribution, which was satisfied for all measures. Levene’s test indicated normal homogeneity of variance for all parameters. A one-way repeated-measures analysis of variance (ANOVA) was performed to clarify the effect of the three shoe CAP conditions on the desired parameters. Any significant differences observed in the ANOVAs were determined through post hoc pairwise comparisons with Bonferroni corrections. The SPSS 25 software (SPSS, Chicago, IL) was used for all statistical analyses, with a significance level of p<0.05.

## Results

Fig 2 shows the average lower limb joint angle during walking in 10-, 30-, and 60 mmHg CAP conditions. The step frequency, step width, and step length were not different between these conditions; however, the stance time significantly decreased as the pressure increased (Table 1). The ankle sagittal ROM in the 60 mmHg condition was significantly lower than those in the 10- and 30 mmHg conditions (p = 0.015). Furthermore, the ankle frontal ROM significantly decreased as pressure increased, although the knee and hip ROM did not differ between conditions. With the increase in CAP, A1, A2, A3, and A4, decreased, whereas A6 increased (p≤0.001). A5 and A7 were not different between 10- and 30 mmHg conditions, although significantly higher values were observed in the 60 mmHg condition. The knee and hip angular parameters were similar across the conditions, except K2, which was significantly higher in the 30 mmHg condition, and H2, which was higher in the 60 mmHg case than those in the 10- and 30 mmHg conditions. The dynamic stability index in the 30- and 60 mmHg conditions was significantly higher than that in the 10 mmHg condition (p = 0.018).

**Table 1:** Spatiotemporal parameters, joint angle, and dynamic stability index when walking with three shoe conditions (mean ± SD).

Variable	10 mmHg	30 mmHg	60 mmHg	p-value
Step frequency (Hz)	107.03 ± 5.55	108.01 ± 6.67	108.14 ± 5.80	**0.204**
Step width (cm)	10.56 ± 3.41	10.49 ± 3.59	10.36 ± 3.55	**0.743**
Step length (cm)	70.13 ± 4.68	70.21 ± 4.32	70.06 ± 3.96	**0.774**
Stance time (s)	0.716 ± 0.032 ^a^^,^^b^	0.706 ± 0.029 ^c^	0.695 ± 0.033	**0.001**
Dynamic stability index	1.18 ± 0.36 ^a^^,^^b^	1.27 ± 0.36	1.29 ± 0.46	**0.018**
Ankle ROM: sagittal (°)	22.78 ± 3.45 ^b^	22.65 ± 3.13 ^c^	21.97 ± 3.51	**0.015**
A1 (°)	3.78 ± 3.78 ^a^^,^^b^	3.13 ± 3.73 ^c^	1.41 ± 3.58	**0.001**
A2 (°)	-2.66 ± 3.81 ^a^^,^^b^	-3.62 ± 4.03 ^c^	-5.13 ± 3.52	**0.001**
A3 (°)	16.37 ± 4.47 ^a^^,^^b^	14.66 ± 4.30 ^c^	12.87 ± 4.21	**0.001**
A4 (°)	-6.44 ± 5.04 ^a^^,^^b^	-7.74 ± 5.09 ^c^	-9.12 ± 4.65	**0.001**
Ankle ROM: frontal (°)	10.38 ± 2.76 ^a^^,^^b^	9.88 ± 3.10 ^c^	9.16 ± 3.12	**0.001**
A5 (°)	1.08 ± 3.83 ^b^	1.14 ± 3.20 ^c^	1.87 ± 3.24	**0.001**
A6 (°)	-4.80 ± 5.28 ^a^^,^^b^	-4.18 ± 4.93 ^c^	-2.89 ± 4.72	**0.001**
A7 (°)	5.60 ± 4.68 ^b^	5.69 ± 4.25 ^c^	6.34 ± 4.66	**0.021**
Knee ROM (°)	67.82 ± 4.29	67.75 ± 4.61	67.76 ± 4.76	**0.914**
K1 (°)	6.75 ± 5.40	6.66 ± 5.42	6.84 ± 5.27	**0.691**
K2 (°)	17.10 ± 8.64 ^a^^,^^b^	18.54 ± 7.36	17.99 ± 8.29	**0.002**
K3 (°)	5.56 ± 5.32	5.61 ± 6.00	5.49 ± 4.78	**0.927**
K4 (°)	70.89 ± 3.57	71.07 ± 3.50	71.15 ± 3.55	**0.555**
Hip ROM (°)	44.71 ± 3.32	44.68 ± 3.43	44.59 ± 3.10	**0.746**
H1 (°)	33.91 ± 6.19 ^a^^,^^b^	34.46 ± 5.98	34.45 ± 6.13	**0.001**
H2 (°)	-5.61 ± 5.36	-5.79 ± 5.42	-5.75 ± 5.20	**0.375**
H3 (°)	39.09 ± 3.63	38.92 ± 3.66	38.88 ± 3.68	**0.473**

a Significant differences between the 10- and 30 mmHg conditions.

b Significant differences between the 10- and 60 mmHg conditions.

c Significant differences between the 30- and 60 mmHg conditions.

**Fig 3:**
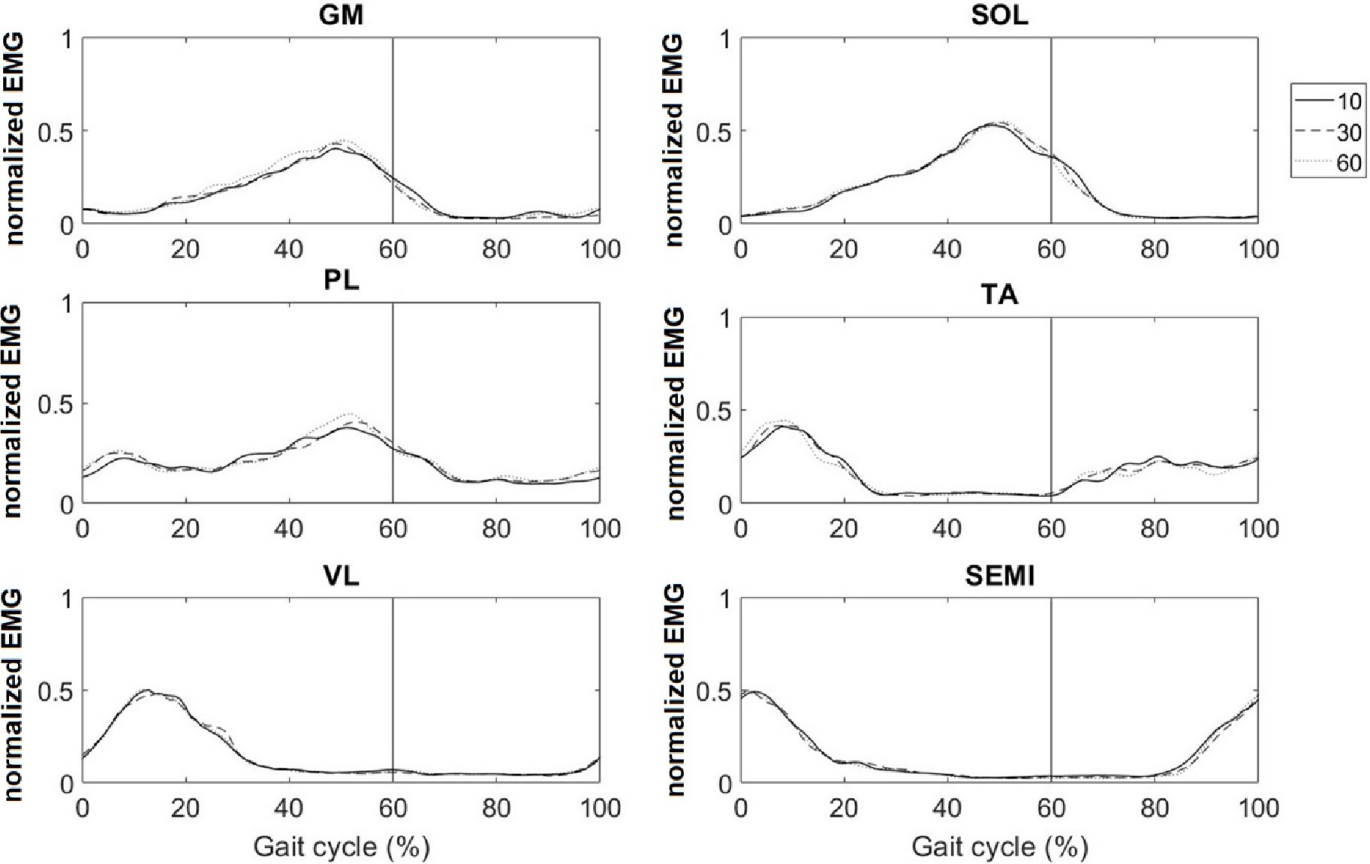
Averaged normalized EMG activity of gastrocnemius medialis (GM), soleus (SOL), peroneus longus (PL), tibialis anterior (TA), vastus lateralis (VL), and semitendinosus (SEMI) muscles when walking with three shoe conditions.

Fig 3 shows the muscular activity for the six studied muscles. During weight acceptance, all ankle muscles exhibited significantly higher activities in the 60 mmHg condition than those in the 10 mmHg condition (Table 2). Comparing 10- to 30 mmHg, the activity of SOL and PL, and comparing 30- to 60 mmHg, the SOL and TA activity was significantly higher. During single limb support, the GM exhibited a higher EMG in the 60 mmHg condition than those in the 30- and 10 mmHg conditions (p = 0.001). During push-off, the activities of all ankle muscles in the 60 mmHg case were higher than that in the 10 mmHg case (p<0.05)). Although their values in the 30- and 60 mmHg cases were similar, the SOL, PL, and TA muscle activities were significantly higher in the 30 mmHg condition compared to the 10 mmHg condition. The VL and SEMI muscles did not differ across the stance phases.

**Table 2:** Muscular activity for four muscles and co-contraction index for antagonistic pairs when walking with three shoe conditions (mean ± SD).

	Weight acceptance				Single limb support				Push-off			
Variable	10 mmHg	30 mmHg	60 mmHg	p-value	10 mmHg	30 mmHg	60 mmHg	p-value	10 mmHg	30 mmHg	60 mmHg	p-value
**Muscular activity**												
GM	6.11 ± 4.31 ^b^	5.88 ± 3.82 ^c^	6.93 ± 6.09	**0.001**	23.52 ± 12.49 ^c^	23.66 ± 12.81 ^c^	25.97 ± 13.94	**0.001**	29.71 ± 16.50 ^b^	31.20 ± 17.60	32.04 ± 19.37	**0.025**
SOL	5.96 ± 3.34 ^a^^,^^b^	6.97 ± 3.89	6.88 ± 4.60	**0.001**	29.21 ± 12.95	29.82 ± 12.90	29.19 ± 13.60	0.230	40.01 ± 15.10 ^a^^,^^b^	42.94 ± 15.40	43.37 ± 14.42	**0.001**
PL	18.76 ± 10.12 ^a^^,^^b^	21.84 ± 13.01	22.30 ± 15.10	**0.001**	23.73 ± 14.52	23.33 ± 13.19	23.66 ± 10.73	0.396	31.86 ± 16.99 ^a^^,^^b^	34.49 ± 16.80	35.11 ± 17.58	**0.012**
TA	35.24 ± 12.30 ^a^^,^^b^	36.15 ± 12.74 ^c^	39.14 ± 14.09	**0.002**	9.43 ± 7.71	9.53 ± 6.97	9.53 ± 5.75	0.977	5.02 ± 2.59 ^a^^,^^b^	5.60 ± 3.94	5.49 ± 3.16	**0.015**
VL	33.61 ± 19.55	34.12 ± 19.95	34.27 ± 18.79	0.087	19.86 ± 8.21	19.10 ± 7.73	19.48 ± 8.07	0.481	5.47 ± 7.76	5.40 ± 3.38	5.61 ± 5.73	**0.252**
SEMI	39.54 ± 14.56	39.11 ± 15.17	39.57 ± 15.45	0.198	7.72 ± 5.73	7.42 ± 5.47	7.23 ± 4.82	0.054	2.93 ± 2.30	2.80 ± 1.69	2.75 ± 1.43	**0.114**
**Co-contraction index**												
GM-TA	0.070 ± 0.047 ^b^	0.068 ± 0.036 ^c^	0.075 ± 0.056	**0.001**	0.062 ± 0.051	0.061 ± 0.043	0.065 ± 0.042	0.067	0.049 ± 0.027 ^b^	0.050 ± 0.029 ^c^	0.058 ± 0.033	**0.001**
SOL-TA	0.071 ± 0.044 ^a^^,^^b^	0.083 ± 0.050	0.083 ± 0.057	**0.001**	0.067 ± 0.050	0.065 ± 0.044	0.070 ± 0.049	0.258	0.050 ± 0.027 ^b^	0.051 ± 0.030 ^c^	0.055 ± 0.031	**0.003**
PL-TA	0.261 ± 0.150 ^a^^,^^b^	0.298 ± 0.185 ^c^	0.322 ± 0.245	**0.001**	0.096 ± 0.073	0.092 ± 0.064	0.095 ± 0.066	0.330	0.053 ± 0.025 ^b^	0.053 ± 0.029 ^c^	0.059 ± 0.030	**0.001**
VL-SEMI	0.309 ± 0.210	0.307 ± 0.195	0.312 ± 0.194	0.804	0.082 ± 0.058	0.084 ± 0.044	0.081 ± 0.050	0.151	0.039 ± 0.024	0.036 ± 0.021	0.037 ± 0.019	**0.091**

a Significant differences between the 10- and 30 mmHg conditions.

b Significant differences between the 10- and 60 mmHg conditions.

c Significant differences between the 30- and 60 mmHg conditions.

The CI for the VL-SEMI pair was similar across conditions in all three parts of the stance phase. During weight acceptance, the CI of all ankle antagonistic pairs was higher in the 60 mmHg case than that in the 10 mmHg case. The corresponding CI for GM-TA and PL-TA for the 60 mmHg condition was higher than that in the 30 mmHg condition. In this period, the CI for SOL-TA and PL-TA pairs was higher in the 30 mmHg cases than that in the 10 mmHg case. The value for all conditions was similar during weight acceptance. During push-off, all three ankle pairs exhibited higher values in the 60 mmHg case than those in the 10- and 30 mmHg conditions, and the values for the 10- and 30 mmHg conditions were comparable.

As indicated in Table 3, the peak pressure in the medial forefoot and lateral rearfoot regions was comparable across conditions. Between the 10- and 60 mmHg conditions, the peak pressure significantly increased for the hallux, lesser toes, lateral forefoot, and midfoot and significantly decreased for the medial rearfoot. Between the 10- and 30 mmHg conditions, the peak pressure of the medial and lateral rearfoot significantly increased and decreased, respectively. The peak pressure for the lateral forefoot and lesser toes regions was significantly different between the 30- and 60 mmHg conditions. Moreover, between the 10- and 60 mmHg conditions, the average pressure significantly decreased for the hallux, lesser toe, and lateral forefoot but increased for the midfoot and lateral rearfoot. The difference in the 30- and 60 mmHg conditions was significant across the four regions, but that between the 10- and 30 mmHg conditions was significant for only the midfoot region. In addition, the impulse for 60 mmHg was significantly higher than those in the 10- and 30 mmHg conditions for all regions except for the hallux. The midfoot and lateral rearfoot impulse was significantly higher in the 30 mmHg case than that in the 10 mmHg. Finally, the rearfoot plantar pressure ratio was significantly higher in the 60 mmHg condition (101.5 ± 18.0, 100.3 ± 20.8, 93.3 ± 19.5; p<0.001 for 10, 30, and 60 mmHg, respectively), indicating that the pressure shifted to the lateral border of the heel in the high CAP condition.

**Table 3:** Plantar pressure parameters when walking with three shoe conditions (mean ± SD).

Plantar region	Conditions (mmHg)	Peak pressure (kPa)	p-value	Average pressure (kPa)	p-value	Impulse (kPa.s)	p-value
Hallux	10	348.8 ± 111.9 ^b^	**0.035**	59.5 ± 17.6 ^b^	**0.001**	44.3 ± 13.5	**0.439**
	30	359.5 ± 109.8		57.2 ± 14.3 ^c^		45.1 ± 13.1	
	60	379.1 ± 126.8		51.0 ± 11.0		43.9 ± 11.3	
Lesser toes	10	234.3 ± 48.2 ^b^	**0.015**	46.9 ± 13.4 ^b^	**0.001**	44.5 ± 17.2 ^b^	**0.001**
	30	238.5 ± 46.6 ^c^		47.2 ± 13.1 ^c^		45.3 ± 17.5 ^c^	
	60	247.7 ± 55.6		43.8 ± 11.0		49.5 ± 16.9	
Lateral forefoot	10	217.9 ± 62.1 ^b^	**0.001**	49.0 ± 13.2 ^b^	**0.002**	79.9 ± 19.9 ^b^	**0.001**
	30	224.5 ± 65.5 ^c^		49.1 ± 14.2 ^c^		81.0 ± 23.5 ^c^	
	60	231.4 ± 65.1		46.8 ± 12.6		87.4 ± 22.1	
Medial forefoot	10	218.5 ± 72.1	0.659	47.7 ± 15.7	0.127	42.8 ± 17.9 ^b^	**0.001**
	30	220.1 ± 71.6		48.8 ± 16.1		44.0 ± 18.9 ^c^	
	60	223.0 ± 81.8		47.2 ± 16.2		48.6 ± 19.2	
Midfoot	10	143.1 ± 32.7 ^a^^,^^b^	**0.001**	31.2 ± 5.5 ^a^^,^^b^	**0.001**	72.8 ± 15.7 ^a^^,^^b^	**0.001**
	30	150.3 ± 26.8		34.8 ± 5.3		77.3 ± 13.6 ^c^	
	60	151.5 ± 30.4		35.4 ± 5.4		85.7 ± 15.5	
Lateral rearfoot	10	242.9 ± 40.2	0.412	59.1 ± 9.4 ^b^	**0.001**	49.6 ± 11.7 ^a^^,^^b^	**0.001**
	30	240.7 ± 45.7		60.7 ± 13.5 ^c^		55.6 ± 14.4 ^c^	
	60	241.5 ± 43.7		65.5 ± 15.1		65.5 ± 15.8	
Medial rearfoot	10	235.9 ± 41.7 ^a^^,^^b^	**0.001**	60.0 ± 13.3	0.360	38.3 ± 10.1 ^b^	**0.001**
	30	228.0 ± 42.0		60.9 ± 14.4		39.1 ± 10.7 ^c^	
	60	228.6 ± 41.5		60.8 ± 13.5		43.6 ± 13.7	

a Significant differences between the 10- and 30 mmHg conditions.

b Significant differences between the 10- and 60 mmHg conditions.

c Significant differences between the 30- and 60 mmHg conditions.

## Discussion

The shoe collar plays a significant role in supporting the ankle during walking. This study represents the first attempt at examining the effect of the collar CAP on the kinematics, dynamic stability, EMG, and plantar pressure during normal walking. Increasing the CAP of the custom-designed shoe resulted in a stiffer collar [20], which provided more support for the ankle by circularly embracing the ankle and shank in a better manner.

An essential biomechanical function of the high-collar is to limit excessive inversion [5], thereby preventing lateral ligament ankle sprains, the most common injury during walking [5, 27]. The findings of this study indicated that with the increase in the collar CAP, the ankle frontal ROM significantly decreased. Moreover, the heel contact (A5) and push-off (A7) inversion peaks increased in the high CAP condition. Compared to the low CAP condition, the medium CAP condition decreased the frontal ROM owing to eversion reduction (A6) without any changes in the inversion peaks. In other words, although both high and medium CAP conditions could restrict the frontal ROM, the former condition put the ankle in a more inverted position. This inverted position persisted in a considerable part of the gait cycle during the high CAP condition (Fig 2). This inappropriate foot positioning may lead to increased susceptibility to ankle sprains [28].

A trade-off exists between the degree of ankle protection provided by a high-collar shoe and ankle ROM [9]. An ideal high-collar shoe must limit the frontal ROM while maintaining the sagittal ROM, which represents the functional ankle ROM. However, to adequately support the ankle in the mediolateral direction, considerable collar stiffness is required [5], which may restrict the ankle sagittal ROM. In this study, the ankle sagittal ROM in the high CAP condition was significantly lower than those in the low and medium conditions. This finding is consistent with those of existing studies, which reported that the ankle sagittal ROM for a stiffer high-collar shoe is smaller than that for a softer high-collar shoe during walking [5–7]. A smaller ankle ROM in the sagittal plane may be associated with inferior shock attenuation performance from ankle dorsiflexion [3]. Additionally, reduced functional ROM of the ankle joint with increased collar stiffness may be associated with decreased power production at the ankle joint, which can adversely influence walking efficiency [7]. Sufficient ankle power is required for forward movement propulsion during gait, and thus, appropriate walking velocities must be achieved [29]. Thus, an optimum collar CAP must not excessively restrict the ankle sagittal ROM.

The knee and hip joint ROM did not differ across CAP conditions. This finding is consistent with those of earlier studies, according to which high-collar footwear limits the ankle but not the knee and hip joints’ kinematics [5–7, 20]. Although significant differences were observed in K3 and H1 across conditions, these parameters did not affect the joint ROM.

Notably, postural stability while walking with high-collar shoes has not been extensively investigated. Researchers used a dynamic stability index similar to that adopted in this study and reported that a high-collar shoe led to higher dynamic stability during walking compared to a normal shoe [1]. Our results showed that increasing the collar CAP to medium and high conditions led to enhanced mediolateral dynamic stability compared to that in the low CAP condition during walking. According to Perry et al. [30], changes in the footwear characteristics affect the mechanical support and interaction between the foot and the environment, potentially limiting the range of COM excursion over the BOS. In our study, the dynamic stability improvement was accompanied by no change in the step width. This finding suggests that increased collar CAP, the only change in shoe properties, might restrict the mediolateral excursion of the COM rather than adjusting foot placement, leading to mediolateral stability improvement. In support, we examined current custom-design shoe during running and found similar results, increased dynamic stability during 30- and 60 mmHg, significantly higher than it was during 10 mmHg condition [20]. Furthermore, it is well established that the application of CAP devices increases the proprioception acuity of the ankle, thereby enhancing static postural stability [17, 31]. The higher CAP conditions of the current high-collar design can be considered to have the same functionality and facilitate somatosensory feedback around the ankle owing to the compression effect. Nevertheless, additional research must be performed to determine whether the enhanced dynamic stability during walking and running is attributable to the mechanical support of the stiffer collar itself or its compression effect.

Collar properties can regulate the amount of muscle activation required to stabilize ankle joints. According to a review study [9], muscle activity increases and decreases with increased and decreased demands on the lower limb, respectively, likely because of the increased mechanical support provided by a high-collar shoe. In contrast, our results indicated higher activity for all ankle muscles during the high CAP condition (in which more support was provided to the ankle) compared to that in the low CAP condition during the weight acceptance and push-off phases. This increase was observed for several ankle muscles as the CAP increased from the low to medium and medium to high conditions, amplifying the demanding effect of CAP on the ankle muscular activity. These contradictory results could be attributed to the design of the studied high-collar shoe: Manipulating the collar CAP using the current shoe design changed not only the collar stiffness but also the proprioceptive inputs that contribute significantly to the enhancement of ankle muscle activation during walking [32]. Nevertheless, the influence of changes in the tactile sensory feedback around the ankle owing to the increased CAP of the high-collar shoes on the ankle muscle activity remains to be clarified. Furthermore, our result indicated no difference in the knee joint muscle activity between the collar conditions. However, Dobson et al. [33] discovered a similar increase in the quadriceps and hamstring muscle activation when participants walked in a stiff shaft boot versus a flexible shaft boot. In addition, Kim et al. [34] reported higher vastus medialis muscle activity during walking with a combat boot compared to that associated with a rain boot. Notably, in these studies, the mass of the boot increased with the increase in the shaft stiffness, whereas the shoe mass did not change between the conditions in this study. Therefore, regardless of changes in footwear support and stiffness, the dominating influence of footwear mass on lower limb motion [35] could be the most likely reason for these contradictory results. This finding suggests that if the mass of the footwear remains constant, the increase in collar stiffness likely does not change the muscular activity of the knee joint.

Although the CI of the VL-SEMI pair did not change across conditions during the stance phase, our results indicated a higher CI for the GM-TA, SOL-TA, and PL-TA pairs with the increase in the collar CAP during the weight acceptance and push-off phases. This increase resulted from the concurrent higher muscular activity of both muscles in the antagonistic pairing. One possible explanation for this phenomenon is the influence of the higher CAP on the ankle position in the weight acceptance and push-off phases. Increasing the collar CAP to the high condition led to a more inverted ankle positioning during weight acceptance (A5 and A6) and push-off (A7) compared to those in the medium and low CAP conditions. In this context, the provision of adequate support by the ankle muscles (especially pronators) when the high-collar shoe forces the foot into a potential ankle sprain situation must be considered [28]. Stronger co-contraction is likely to increase the compression forces inside the joint, thereby increasing the stability [25]. Therefore, increased inversion introduced by the high CAP condition likely triggered the neuromuscular and co-contraction response of the ankle antagonistic muscle pairs to stabilize the ankle. In contrast to our findings, Böhm and Hösl [5] reported significantly increased CI for the VL-SEMI pair for a hard boot shaft compared to a soft boot shaft during single limb support of walking. Moreover, the GM-TA and PL-TA pairs exhibited comparable CI values in the stance phase. Notably, except for the difference in the footwear design and methodology, the authors [5] did not provide information regarding the amount of muscular activity of the antagonistic pair, which renders it challenging to compare the results of the two studies.

In general, the effect of changes in the collar parameters, e.g., the height and stiffness, on the plantar pressure during walking has not been clarified. This study is the first to provide information regarding the influence of high-collar CAP on plantar pressure. According to our results, between the low and high CAP conditions, the peak pressure significantly increased for the hallux, lesser toes, lateral forefoot, and midfoot, whereas it significantly decreased for the medial rearfoot. This increase was notable for the lateral forefoot and lesser toes, especially in medium to high CAP conditions. The inverted position of the foot can likely explain this finding during the stance phase of the high CAP condition, which led to a higher peak pressure across the lateral border of the foot. Further, between the high and other CAP conditions, the average pressure significantly decreased for the hallux, lesser toe, and lateral forefoot and increased for the lateral rearfoot. Unexpectedly, all regions except the hallux exhibited a significantly higher impulse in the high CAP condition compared to those in the medium and low CAP conditions, which indicated that the average pressure was applied over a smaller period. This finding was supported by decreased stance time in the high CAP condition. Furthermore, a lower rearfoot plantar pressure ratio was observed in the high CAP condition, indicating increased relative pressure under the lateral aspect of the heel. This phenomenon is expected, given that an inverted foot position was observed at the heel strike (A5) with a high CAP, which likely resulted in a pressure shift to the lateral rearfoot. A decreased mediolateral rearfoot pressure ratio has been linked to lateral ankle sprains [36], indicating that a high CAP may deteriorate the walking biomechanics. A similar result has been found for testing current high-collar shoe during running [20].

### Limitations

For certain participants, the medial or lateral malleolus ankle markers had to be placed directly on the pressure ratcheting reels owing to the shoe design. In these cases, we created a virtual marker in Visual3D software. For this purpose, the distance between the marker and the conventional landmark of the ankle joint (tip of the malleolus) was measured. Then a virtual marker was created using this distance and used for static and dynamic analysis. Moreover, the findings must be interpreted with caution in real-life applications because they pertain only to the considered shoe design.

## Conclusions

High and medium CAP conditions restricted the inversion-eversion ROM compared to that in the low CAP condition. However, the high CAP condition hindered the sagittal ROM, which can adversely influence walking biomechanics. Similar dynamic stability improvements were observed for high and medium CAP, higher than that in the low CAP condition, indicating the positive effect of a high collar with high CAP. The CAP placed higher demands on the ankle muscular activity, especially in the weight acceptance and push-off phases of walking. This phenomenon is attributable to the higher collar stiffness or enhanced proprioception that needs further investigation. The knee and hip joint ROM were similar across the CAP conditions, which may indicate that the effect of wearing a high-collar shoe on walking angular kinematics is limited to the ankle joint. A higher relative pressure was observed under the lateral aspect of the heel when walking with the high CAP condition, which has been associated with lateral ankle sprains. Together, these results suggest that a high-collar shoe with a high CAP may not be an appropriate choice for walking, owing to the injury risk factors and limited walking efficiency. A medium CAP may provide certain advantages, rendering it a superior choice for a high-collar shoe design. The presented findings can provide guidance for the design and purchase of high-collar shoes.

## Supporting information

S1 Dataset (XLSX)
