# Correction: Influence of circumferential ankle pressure of shoe collar on the kinematics, dynamic stability, electromyography, and plantar pressure during normal walking

**DOI:** 10.1371/journal.pone.0318305

**Published:** 2025-01-24

**Authors:** The PLOS ONE Staff

The fifth author’s initials appear incorrectly in the citation. The correct citation is: Nasirzadeh A, Yang S-T, Yun J, Yang J, Bae YY, Park J, et al. (2023) Influence of circumferential ankle pressure of shoe collar on the kinematics, dynamic stability, electromyography, and plantar pressure during normal walking. PLOS ONE 18(2): e0281684. https://doi.org/10.1371/journal.pone.0281684.

The publisher apologizes for the error.
